# Naked mole-rats maintain cardiac function and body composition well into their fourth decade of life

**DOI:** 10.1007/s11357-022-00522-6

**Published:** 2022-02-02

**Authors:** Emine Can, Megan Smith, Bastiaan J. Boukens, Ruben Coronel, Rochelle Buffenstein, Johannes Riegler

**Affiliations:** 1 Calico Life Sciences LLC, 1170 Veterans Blvd, South San Francisco, CA 94080 USA; 2 Department of Medical Biology, Amsterdam University Medical Centers, University of Amsterdam, 1105 AZ Amsterdam, The Netherlands; 3 Department of Physiology, Cardiovascular Research Institute Maastricht, Maastricht University Medical Center, 6200 MD Maastricht, The Netherlands; 4 Department of Experimental Cardiology, Heart Center, Academic University Medical Centers, University of Amsterdam, 1105 AZ Amsterdam, The Netherlands; 5 Present Address: Department of Biology, University of Illinois at Chicago, Chicago, IL 60607 USA

**Keywords:** Aging, Cardiac function, Echocardiography, Magnetic resonance imaging, Electrocardiogram, Naked mole rat, Mouse, Arrhythmia

## Abstract

The prevalence of cardiovascular disease increases exponentially with age, highlighting the contribution of aging mechanisms to cardiac diseases. Although model organisms which share human disease pathologies can elucidate mechanisms driving disease, they do not provide us with innate examples how cardiac aging might be slowed or attenuated. The identification of animal models that preserve cardiac function throughout most of life offers an alternative approach to study mechanisms which might slow cardiac aging. One such species may be the naked mole-rat (NMR), a mouse-sized (40 g) rodent with extraordinary longevity (> 37 years), and constant mortality hazard over its four decades of life. We used a cross-sectional study design to measure a range of physiological parameters in NMRs between 2 and 34 years of age and compared these findings with those of mice aged between 3 months and 2.5 years. We observed a rapid decline in body fat content and bone mineral density in old mice, but no changes in NMRs. Similarly, rhythm disorders (premature atrial and ventricular complexes) occurred in aged mice but not in NMRs. Magnetic resonance and ultrasound imaging showed age-dependent increases in cardiac hypertrophy and diastolic dysfunction in mice which were absent in NMRs. Finally, cardiac stress tests showed an age-dependent decline in normalized cardiac output in mice, which was absent in NMRs. Unlike mice, that manifest several aspects of human cardiac aging, NMRs maintain cardiac function and reserve capacity throughout their long lives and may offer insights on how to delay or prevent cardiac aging.

**Supplementary Information:**

The online version contains supplementary material available at 10.1007/s11357-022-00522-6.

## Introduction

With aging human populations cardiovascular diseases have become the primary cause of death globally and a substantial burden for healthcare systems [1]. Indeed, age is the strongest risk factor for cardiovascular diseases [2]. Furthermore, the prevalence of many comorbidities such as obesity, dyslipidemia, and diabetes also increases substantially with age compounding the severity of cardiovascular pathology and mortality [3]. Mice manifest many aspects of cardiac aging that are commonly observed in humans. For example, cardiac hypertrophy, diastolic dysfunction, increased rates of arrhythmia, and decreased functional reserve capacity become evident at less than half of their observed maximum lifespan [4–8]. Not surprisingly, mice have been routinely used as experimental models for understanding the molecular mechanisms controlling these age-related changes and for the development of disease modifying therapies. However, living only half as long as predicted on the basis of body size and having naturally poor defenses against aging, mice may not necessarily elucidate protective mechanisms to attenuate or abrogate the progression of age-associated cardiovascular disease. An alternative approach would be to find an animal model that may naturally resist these processes, exploiting the mechanisms that evolution through millions of years of natural selection has already discovered and thereby learn by example how to prevent, delay, or retard the effects of aging on cardiovascular function. The quest to identify such a model has, however, remained elusive because aging is considered by many to be inevitable and unmodifiable.

The naked mole-rat (*Heterocephalus glaber*; NMR) [9] may harbor the mechanisms we are trying to emulate in successfully retarding cardiac aging. These small rodents (30–50 g) live far longer (> 37 years) than predicted allometrically, and exceed their expected lifespan based upon various developmental milestones (e.g., age at sexual maturity). Moreover, they maintain good health [10] and a low mortality hazard throughout their long lives [11]. Rather, unlike the exponentially increasing mortality hazard observed in humans and other mammals at ages three–four fold greater than the age of sexual maturity, the mortality hazard of NMRs remains constant to ages at least 27-fold greater than sexual maturity and death appears to be stochastic [11]. A constant mortality hazard implies an exceptional ability to maintain homeostasis of major organ systems with advancing age and supports the premise of a lack of functional aging. Essentially, these rodents can be considered an excellent mammalian model of successful aging*.*

Previous studies using younger animals have indicated that NMRs do not show age associated cardiac hypertrophy, diastolic dysfunction, or a decline in functional cardiac reserve up to 24 years of age [12, 13] nor do their blood vessels lose their elasticity in middle age [14]. However, a comprehensive comparison of changes in cardiovascular function as well as other physiological parameters across the lifespan of NMRs and mice is lacking. Using a variety of imaging techniques, we set out to compare changes in cardiac function, functional reserve capacity, cardiac electrophysiology, body composition, and bone mineral density in NMRs and mice across their respective lifespans. These new studies confirm pronounced species differences in age-related changes in body composition and cardiac properties and provide strong support for using the naked mole-rat as an animal model for successful cardiac aging.

## Methods

All animal studies were approved by either Calico’s or the Buck Institute’s Institutional Animal Care and Use Committee (IACUC).

### Mice

Both sexes *of CD57BL/6J* were purchased from the Jackson Laboratory at 10–90 weeks of age and habituated to our facilities where they were housed until used for our experiments. Mice were housed on a 12-h light/dark cycle (light 06:00–18:00) in individually ventilated cages with a room temperature of 19–20 °C and a humidity of 20–30%. Mice had ad libitum access to water and standard chow (LabDiet 5001) and were housed in cages of up to five animals of the same sex. Separate cohorts were enrolled in this study at 3, 20, 28, and 30 months of age (*n* = 62 females and *n* = 73 males).

### Naked mole-rats

Animals of both sexes and ranging in age between 2 and 34 years were employed in this study (*n* = 48 females and *n* = 97 males). These NMRs were part of the well characterized Calico colony. Animals were housed in climatically controlled rooms maintained at 28–30 °C, 30–50% relative humidity, on a 12-h light/dark cycle (light 06:00–18:00) and kept in multi-chambered plexiglass burrow systems. They were fed ad libitum with yams as well as a selection of other fruit and vegetables (bananas, apples, oranges, butternut squash, red bell pepper, romaine lettuce, cucumber, green beans, corn, carrots) and supplemented weekly with a high protein and vitamin enriched cereal (Pronutro, South Africa). Drinking water was not provided, rather NMRs meet all their water requirements from their diet. All animals are sexed and microchipped at 90 days of age, providing unique nine-digit animal identifiers.

### Anesthesia

Both species were anesthetized with isoflurane (Henry Schein, NY; anesthesia induction: mice 4% and NMR 5%, anesthesia maintenance: mice: 1–2%; NMR 1.5–2.5%) and placed onto an animal cradle or platform. During data acquisition, animals were kept at their respective body temperature (mice; 37 ± 0.4 °C; NMR; 34 ± 0.5 °C) via a water heated pad and an air heating system (MRI) or heated platform (ultrasound, ECG), while medical air and anesthetics (isoflurane) were supplied via a nose cone (0.4 L/min). Isoflurane levels were adjusted to maintain a breathing rate of 50–80 breaths/min in mice and 40–50 breaths/min in NMRs. Animals were imaged in a supine or prone position. Dual energy X-ray absorption (DEXA) imaging was performed without heating.

### Electrocardiogram recordings

Anesthetized mice were positioned supine on a heated platform (Indus Instruments, Texas), and subcutaneous needle electrodes were positioned in the four limbs. Anesthetized NMRs were positioned prone with their four limbs resting on surface electrodes covered with contact gel. Once body temperature and breathing rates had stabilized in the specified range (see above) five minutes of ECG recordings were saved. Recordings from the ECG traces (L1, L2, L3), temperature and breathing were exported and analyzed using an in-house Matlab script. One hundred ECG traces were averaged for ECG pattern analysis. Potentially irregular heartbeats were identified by R-R intervals which were < 70% or > 130% of the moving average R-R interval. Potential irregular heartbeats were manually classified based on the three-lead ECG, by two independent experts as no arrhythmia, atrial premature beat, ventricular premature beat, or junctional premature beat.

### Dual energy X-ray absorption imaging

Anesthetized mice and NMRs were positioned prone on the X-ray detector of a rodent DEXA imaging system (Piximus, GE Lunar, WI). While mice were covered in a single imaging sequence, two imaging sequences were required for NMRs with the first covering the upper body and the second the lower body. Images were analyzed with the vendor software including manual adjustments for head exclusion masks and positioning of femur region of interest masks. Results from the two image sequences of each NMR were combined. Quality control was performed daily following manufacturer’s instructions and the system re-calibrated if quality control criteria were not met.

### Magnetic resonance imaging

Imaging was performed using a preclinical 9.4 T (BioSpec 94/20 USR) horizontal bore scanner (Bruker, Ettlingen, Germany) with a shielded gradient system (660 mT/m). Anesthetized animals were positioned prone in an animal cradle. An animal monitoring system (Small Animal Instruments, NY) consisting of a fiberoptic temperature sensor, subcutaneous needle electrodes (ECG), and a breathing pad were used to monitor animals and provide gating signals. Data acquisition was performed with a 4-channel phased array receive only surface coil (Bruker) placed around the heart and centered in a decoupled 86-mm transmit/receive volume coil (Bruker). Long- and short-axis scout images were acquired to define the two- and four-chamber long-axis views. The cine long-axis views were used to define short-axis slice stacks. A prospectively double-gated (ECG and respiration) spoiled gradient echo sequence with flow compensation was used to acquire cine cardiac images using the following parameters for mice: TE 1.8 ms, TR 4.5–6 ms, flip angle 16°, slice thickness 0.8 mm, no slice gap, FOV 24 × 24 mm^2^, matrix size 192 × 192, NSA 1 for short-axis, and 2 for long-axis. Twenty cine-frames were recorded to cover the cardiac cycle. A single short-axis slice was obtained in approximately 50 s, leading to a total scan time of 10 min covering the heart from base to apex (12 slices) in mice. After the baseline data set (2-ch, 4-ch, short-axis stack) was acquired, dobutamine hydrochloride 1.5 mg/kg diluted in saline (200 μg dobutamine/ml) was infused through an intraperitoneal catheter at a rate of 1 ml/min. A second short axis-imaging stack (dobutamine stress) was acquired after a wait period of 6 min to achieve sufficient dobutamine uptake in the heart. The imaging protocol for one mouse typically lasted 45 min. While the same imaging protocol was used for NMRs but to accommodate their lower heart rate, TR and flip angle were increased to 10–20 ms and 20–25°, respectively. Anonymized data sets were analyzed using a semi-automatic segmentation software, Segment v3.2 R8402 (http://segment.heiberg.se, Medviso AB, Sweden) as previously described [15, 16]. Left atrial volumes were estimated from 2-ch and 4-ch long-axis cine using the biplane method.

### Ultrasound imaging

Ultrasound imaging was performed using a linear transducer (MX550D) with a center frequency of 40 MHz connected to a Vevo 3100 ultrasound system (Visualsonics, Toronto, Canada).

To measure baseline cardiac function in NMRs, electrocardiogram kilohertz visualization (EKV) gated parasternal long axis view images were acquired followed by Doppler transmitral flow measurements, EKV gated images of the abdominal aorta, and Doppler measurements of abdominal aorta blood flow. Once baseline imaging was completed, dobutamine hydrochloride 1.5 mg/kg diluted in saline (200 μg dobutamine/ml) was injected intraperitoneally using a 31G insulin syringe. Six minutes after dobutamine injection a second set of EKV gated parasternal long axis views was acquired (dobutamine stress test). The imaging protocol required approximately 25 min per NMR.

To assess diastolic function and abdominal aorta dimensions, animals were imaged in supine position. Data analysis including calculation of the LV systolic and diastolic function was performed off-line with the use of a commercially available software (VevoLab, VisualSonics) by a blinded observer. *E*/*A* ratios were only quantified when early and atrial inflow peaks were clearly separable.

### Statistical analysis

To test for possible correlations between functional parameters and time, linear models with time, time^2^, and sex as explanatory variables were tested. The model with the smallest number of parameters that fitted the data best was selected. A Wilcoxon ranked sum test was used to test for differences between specified cohorts of both species. Statistical analysis was performed using R software version 3.5.1.

## Results

### Naked mole-rat body composition changes minimally with advancing age.

**Fig. 1:**
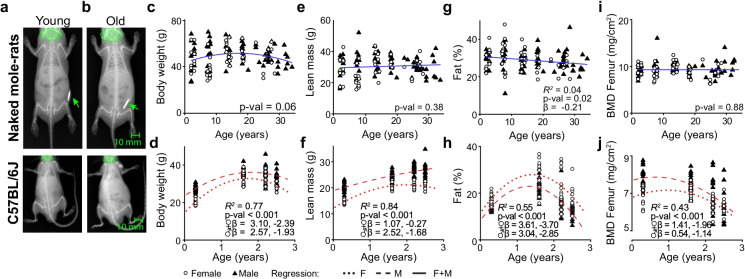
Unlike mice, NMR body composition and bone mineral density show minimal age-related changes. **a**, **b** Representative X-ray images of young and old NMRs as well as mice (NMR: 3 and 26.5 years; mouse: 3 months and 2.5 years). Arrows indicate radio frequency identification chips, while green circles illustrate regions of interest used to exclude heads from the analysis. **c** NMR body weight showed a small quadratic age dependence (*n*: ♀ = 48 ♂ = 72, Age *β* = 0.87, Age^2^ *β* =  − 0.85, *P* = 0.06). **d** Mouse body weight showed a strong quadratic age dependence (*n*: ♀ = 62 ♂ = 70, Age ♀*β* = 3.10, Age^2^ ♀*β* =  − 2.39, *P* < 2.2E − 16, Age ♂*β* = 2.57, Age^2^ ♂*β* =  − 1.93, *P* < 2.2E − 16). **e** NMR lean body mass (excluding head) did not change with age (*n*: ♀ = 48 ♂ = 72, Age *β* = 0.08, *P* = 0.38). **f** Mouse lean body mass (excluding head) started to decline at 2 years of age for females and later for males (*n*: ♀ = 62 ♂ = 73, Age ♀*β* = 1.07, Age^2^ ♀*β* =  − 0.27 *P* < 2.2E − 16, Age ♂*β* = 2.52, Age^2^ ♂*β* =  − 1.68, *P* < 0.001). **g** NMR body fat fraction declined 12% over 33 years (*n*: ♀ = 48 ♂ = 72, Age *β* =  − 0.21, *P* = 0.02). (h) Mouse body fat fraction declined after peaking around 1.5 years of age in females and males (*n*: ♀ = 62 ♂ = 70, Age ♀*β* = 3.61, Age^2^ ♀*β* =  − 3.70, *P* < 0.001, Age ♂*β* = 3.04, Age^2^ ♂*β* =  − 2.84, *P* < 0.001), with the average being 41% lower in the oldest cohort when compared to the 18-month-old cohort. **i** NMR femur bone mineral density (BMD) did not change with age (*n*: ♀ = 48 ♂ = 72, *A*ge *β* = 0.01, *P* = 0.87). **j** Mouse femur bone mineral density started to decline after 1.5 years of age in females and males (*n*: ♀ = 62 ♂ = 70, Age ♀*β* = 1.41, Age^2^ ♀*β* =  − 1.96, *P* = 2.1E − 7, Age ♂*β* = 0.54, Age^2^ ♂*β* =  − 1.14, *P* = 3.1E − 8). Scale bars: 10 mm

We used dual energy X-ray (DEXA) to assess changes in lean mass, fat mass, and bone mineral density at various time points distributed over the expected lifespan of naked mole-rats and C57BL6/J mice (Fig. 1a, b). Body weight showed a small quadratic age dependence with no difference between male and female NMRs in contrast to mice which showed a strong age dependence and sex difference (Fig. 1c, d). While young (2 years) and old (> 30 years) NMRs had a similar bodyweight (46.9 ± 11.3 g and 45.7 ± 7.8 g), mouse body weight increased slightly with age. Lean body mass did not change significantly with age in NMRs (Fig. 1e), while in C57BL6/J mice, lean mass showed a linear increase for males and a nonlinear decline starting at two years of age for females (Fig. 1f).

A small linear decline in body fat fraction was observed in NMRs with advancing age leading to a 12% decline over 33 years (Fig. 1g; Sup. Fig. S1a). In contrast, regardless of sex, mouse body fat fraction declined significantly after 1.5 years of age with a 41% loss in the oldest cohort (2.5 years) compared to 1.5 years old mice (Fig. 1h; Sup. Fig. S1b). The loss of fat mass is primarily responsible for the decline in the body weight of older mice.

Regardless of sex, NMR femur bone mineral density did not change with age (Fig. 1i; Sup. Fig. S1c), whereas in both male and female mice, femoral bone density started to decline after 1.5 years of age (Fig. 1j; Sup. Fig. S1d).

### Naked mole-rat QRS, PR, and PQ duration do not increase with age

**Fig. 2:**
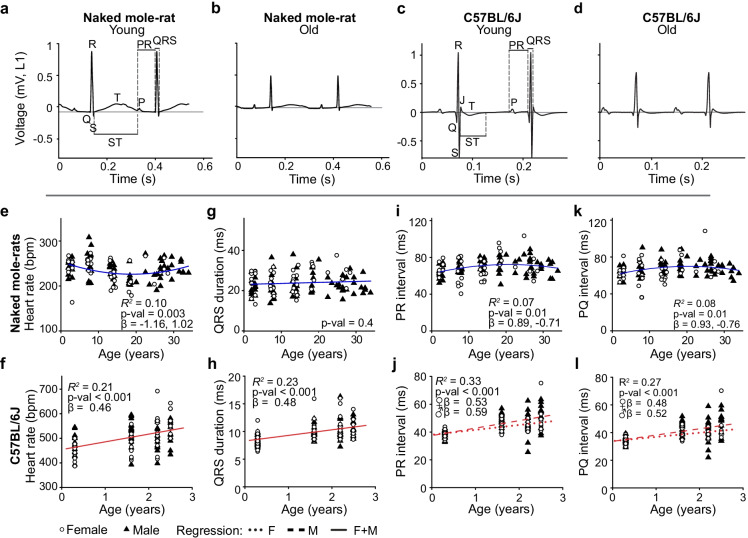
Naked mole-rat QRS, PR, and PQ duration do not increase with age. **a**, **b** Representative ECG traces (average of 100 heartbeats, lead 1) of young (3 years) and old (32.5 years) male NMRs. **c**, **d** Representative ECG traces of young (0.3 years) and old male (2.5 years) mice. **e** Heart rates (under light anesthesia) of NMRs showed a quadratic age dependence (*n*: ♀ = 48 ♂ = 72, Age *β* =  − 1.16, Age^2^ *β* = 1.02, P = 2.9E − 3). But heart rates were similar for the youngest and oldest cohorts (242 ± 23 and 238 ± 13 bpm, *P* = 0.43). **f** Heart rates of mice (under light anesthesia) increased linearly with age (*n*: ♀ = 62 ♂ = 70, *β* = 0.46, *P* = 3.7E − 7). **g** NMR QRS duration did not change with age (*n*: ♀ = 48 ♂ = 72, Age *β* = 0.08, *P* = 0.37). **h** Mouse QRS duration increased linearly with age (*n*: ♀ = 62 ♂ = 70, *β* = 0.48, *P* = 4.63E − 9). **i** NMR PR intervals were quadratically age dependent (*n*: ♀ = 48 ♂ = 72, Age *β* = 0.89, Age^2^ *β* =  − 0.71, *P* = 0.01). **j** Mouse PR intervals increased linearly with age with a small sex difference (*n*: ♀ = 62 ♂ = 70, Age ♀*β* = 0.53, *P* = 1.3E − 5, ♂*β* = 0.59, *P* = 7.8E − 8). **k** NMR PQ intervals were quadratically age dependent (*n*: ♀ = 48 ♂ = 72, Age *β* = 0.93, Age^2^ *β* =  − 0.76, *P* = 0.01). **l** Mouse PQ intervals increased linearly with age with a small sex difference (*n*: ♀ = 62 ♂ = 70, Age ♀*β* = 0.48, *P* = 8.1E − 5, ♂*β* = 0.52, *P* = 4.7E − 6)

To assess whether aging leads to altered cardiac conduction in NMRs and mice, we recorded five minutes of three-lead electrocardiography data and averaged 100 heartbeats for analysis (Fig. 2a–d). NMR heart rate showed a quadratic age dependence (Fig. 2e). However, there was no difference between the youngest and oldest cohorts (242 ± 23 and 238 ± 13 bpm). Mouse heart rates increased significantly with age (Fig. 2f).

NMR QRS duration did not change with age (Fig. 2g), while mouse QRS duration increased linearly with age from 8.5 ± 1.1 ms at three months of age to 10.5 ± 1.5 ms at 2.5 years of age (Fig. 2h). Similarly, no differences were found for PR and PQ intervals between young and old NMRs in contrast to mice where both parameters increased linearly with age (Fig. 2i–l). Age associated PR and QRS interval increases in mice suggest atrio-ventricular and intra-ventricular conduction delays indicative of conduction system aging which was not observed in NMRs

### Arrhythmia frequency increases with age in mice

**Fig. 3:**
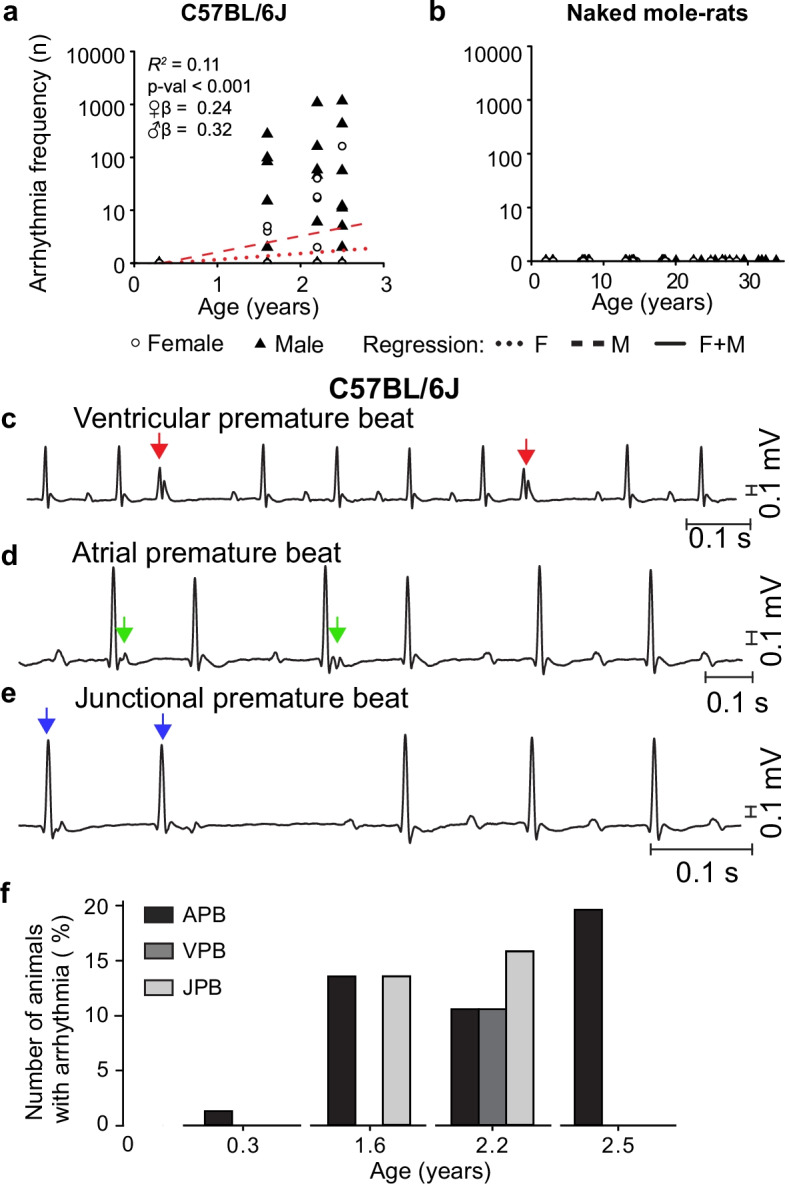
Arrhythmia frequency increases with age in mice. **a** Arrhythmia frequency increases with age in mice with a slightly faster rate in males (*n*: ♀ = 62 ♂ = 70, Age ♀*β* = 0.24, *P* = 0.05, Age ♂*β* = 0.32, *P* = 0.01). **b** No arrhythmias were detected in naked mole-rats (*n*: ♀ = 48 ♂ = 72). **c**–**f** Representative Lead-I ECG traces from 2.2-year-old male mice showing commonly observed arrhythmias: atrial premature beat (APB) indicated by red arrows, ventricular premature beat (VPB) indicated by green arrows, and junctional premature beat (JPB) indicated by blue arrows. **g** Percentage of mice with different arrhythmia types across the observed age range (*n*: ♀ = 62 ♂ = 70)

Five minutes of three-lead electrocardiography data were screened for irregular heartbeats which were manually classified as normal or one of three arrhythmia types by two experts. Both the number of animals with arrhythmias and their frequency within the recording period increased with age in male and female mice (Fig. 3a). Interestingly, no arrhythmias were found in NMR ECG recordings (Fig. 3b). Examples of the three arrhythmia types detected in mice older than 1.5 years are shown in Fig. 3c–e. The most prevalent conduction abnormality at all ages in mice was atrial premature contraction, reaching a prevalence of 20% for mice older than 2.2 years (Fig. 3g). Other frequently observed arrhythmias were ventricular premature beats and junctional premature beats.

### Naked mole-rat cardiac function at rest does not change with age

**Fig. 4:**
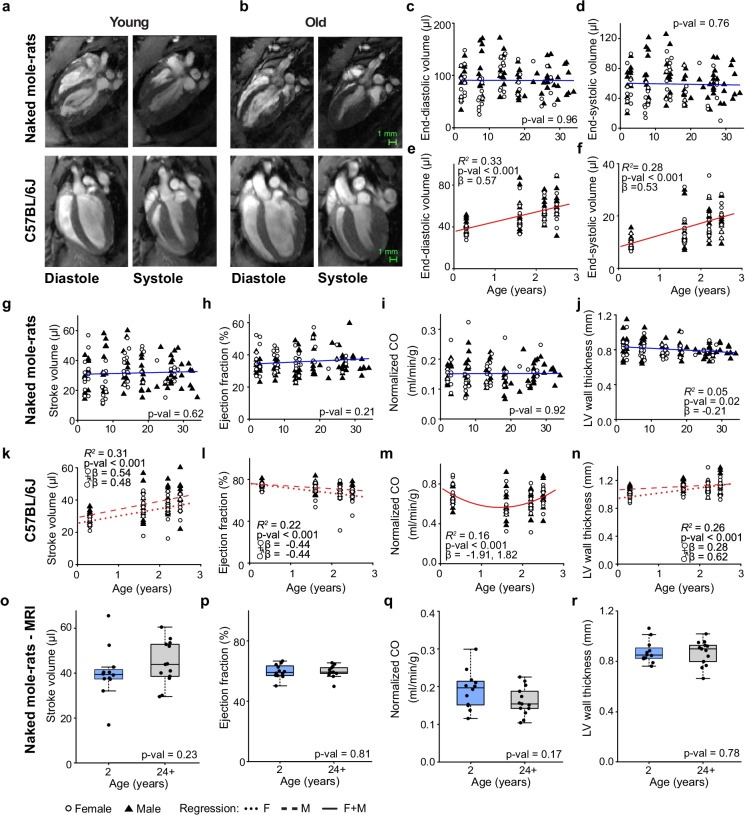
NMR cardiac function at rest does not change with age. **a**, **b** Representative magnetic resonance 4-chamber long axis images of a young (2 years) and old (32 years) NMR heart as well as young (0.3 years) and old (2.5 years) mouse heart at end-diastole and end-systole. **c**, **d** NMR end-diastolic volume and end-systolic volumes (ultrasound) did not change with age (*n*: ♀ = 48 ♂ = 72, Age *β* =  − 4.4E − 3, *P* = 0.96 and Age *β* =  − 0.03, P = 0.76). **e**, **f** Mouse end-diastolic and end-systolic volumes increased linearly with age (*n*: ♀ = 47 ♂ = 49, Age *β* = 0.57, *P* = 1.2E − 9 and Age *β* = 0.53, *P* = 2.9E − 8). **g**, **h** NMR left ventricular stroke volumes and ejection fraction (ultrasound) did not change with age (*n*: ♀ = 48 ♂ = 72, Age *β* = 0.05, *P* = 0.62 and Age *β* = 0.11, *P* = 0.21). (i) NMR cardiac output (ultrasound) normalized to body weight did not change with age (*n*: ♀ = 48 ♂ = 72, Age *β* = 0.01, *P* = 0.93). **j** NMR left ventricular (LV) anterior wall thickness (ultrasound) at end-diastole showed a small age associated decline (*n*: ♀ = 48 ♂ = 72, Age *β* =  − 0.21, *P* = 0.02). **k**, **l** Mouse left ventricular stroke volume increased with age while ejection fraction declined linearly with age (*n*: ♀ = 47 ♂ = 49, Age ♀*β* = 0.54, *P* = 7.7E − 5, Age ♂*β* = 0.48, *P* = 4.6E − 4 and Age ♀*β* =  − 0.44 P = 2.1E − 3, Age ♂*β* =  − 0.44, *P* = 1.5E − 3). **m** Mouse cardiac output normalized to body weight showed a quadratic age dependency (*n*: ♀ = 47 ♂ = 49, Age *β* =  − 1.91, Age^2^ *β* = 1.82, *P* = 3.3E − 4), but there was no difference between the youngest and oldest cohort (Wilcoxon, *P* = 0.89). **n** Mouse left ventricular mid anterior wall thickness at end-diastole increased linearly with age (*n*: ♀ = 47 ♂ = 49, Age ♀*β* = 0.62, *P* = 2.82E − 6, Age ♂*β* = 0.28, *P* = 0.05). **o** Young and old male NMR left ventricular stroke volume (MRI) were not significantly different (*n*: ♂ = 12 ♂ = 13, Wilcoxon, *P* = 0.23). **p**, **q** NMR left ventricular ejection fraction and normalized cardiac output (MRI) of young and old NMRs were not significantly different (*n*: ♂ = 12 ♂ = 13, Wilcoxon, *P* = 0.81 and *P* = 0.17). **r** Young and old NMR left ventricular wall thicknesses (MRI) at end-diastole were not significantly different (*n*: ♂ = 12 ♂ = 13, Wilcoxon, *P* = 0.78). LV: left ventricular, scale bars 1 mm

To determine the influence of age on cardiac anatomy and cardiac function at rest, we investigated systolic and diastolic characteristics of both species using echocardiography and MRI. Figure 4a and b show representative end-diastolic and end-systolic long-axis images of young and old NMRs as well as C57BL/6J mouse hearts. We used ultrasound imaging (Sup. Fig. S4a) to assess cardiac function in NMRs since their radio frequency identification chips (RFIDs) were often close to the heart leading to substantial signal loss on MR images.

Left ventricular end-diastolic and end-systolic volumes remained constant with increasing age in NMRs (Fig. 4c and d). In contrast to NMRs, a linear increase in end-diastolic and end-systolic volumes with age was observed in mice (Fig. 4e, f).

NMR left ventricular stroke volumes and ejection fractions did not change with age (Fig. 4g, h), neither did normalized cardiac output (Fig. 4i; Sup. Fig. S4b). Finally, NMR left ventricular anterior wall thickness showed a small age associated decline (Fig. 4j). Unlike NMRs, mouse left ventricular stroke volumes increased with age, while ejection fractions declined linearly with age (Fig. 4k, l). The age associated left ventricular ejection fraction decline observed in mice was due to a faster age associated increases in end-diastolic volumes compared to end-systolic volumes. Normalized mouse cardiac output showed a quadratic age dependency without significant differences between the youngest and oldest cohorts (Fig. 4m; Sup. Fig. S4c). Mouse left ventricular mid anterior wall thickness increased linearly with age (Fig. 4n; Supp. Fig. S4d-g).

To validate our ultrasound-derived measurements, cardiac MRI was performed on a different cohort of young and old male NMRs whose RFID chips were sufficiently distant from the heart (> 4 cm) to enable MRI. The mean age of the young and old cohort was 1.9 ± 0.1 and 28.2 ± 2.3 years, respectively, while body weights were similar (Supp. Fig. S4l). Ventricular volumes and cardiac function at rest were similar between young and old NMRs (Fig. 4o–q; Sup. Fig S4h-i). No differences in left ventricular wall thickness between young and old NMRs were observed (Fig. 4r; Sup. Fig. S4j, k), highlighting the absence of age-associated cardiac hypertrophy.

### Diastolic function did not change with age in naked mole-rats

**Fig. 5:**
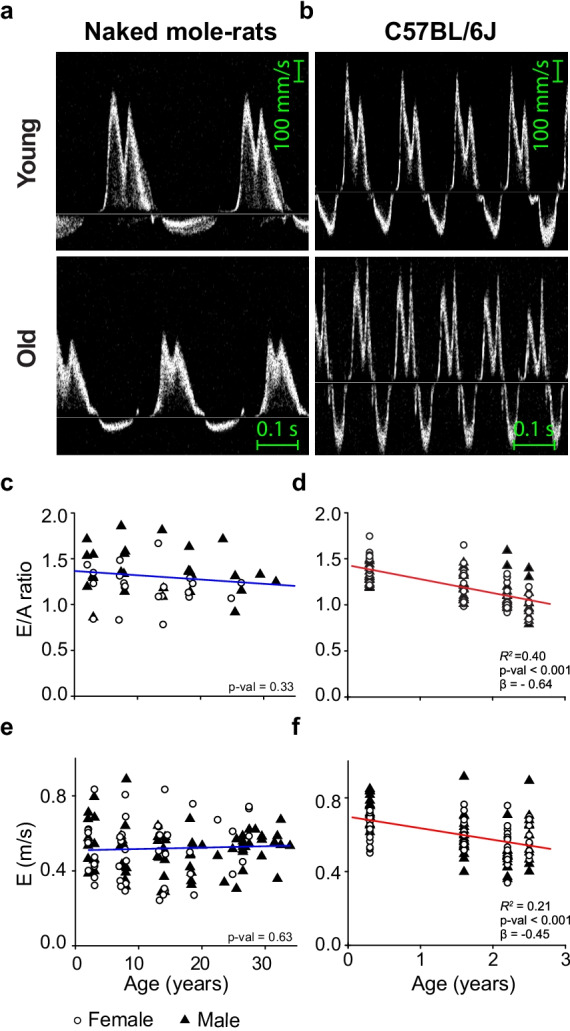
NMRs did not show age associated diastolic dysfunction. **a**, **b** Representative Doppler left ventricular flow measurements in a young (2 years) and old (26.5 years) NMR as well as young (0.3 years) and old (2.5 years) mouse heart. **c** NMR early/atrial (*E*/*A*) filling ratios did not change with age (*n*: ♀ = 22 ♂ = 29, Age *β* =  − 1.14, *P* = 0.33). **d** Mouse *E*/*A* ratios declined linearly with age (*n*: ♀ = 52 ♂ = 61, Age *β* =  − 0.64, *P* = 5.9E − 14). **e** NMR early peak filling velocities did not change with age (*n*: ♀ = 48 ♂ = 71, Age *β* = 0.05, *P* = 0.63). **f** Mouse early peak filling velocities declined linearly with age (*n*: ♀ = 52 ♂ = 61, Age *β* =  − 0.45, *P* = 4.1E − 7). E: early filling velocity, A: atrial filling velocity, Scale bars 0.1 s

To investigate the age dependency of diastolic function in NMRs and mice, we measured the ratio of early to atrial inflow velocity (*E*/*A*) through the mitral valve. Doppler mitral flow patterns from representative young (2 years) and old (26.5 years) NMR hearts as well as a young (three months) and old (2.5 years) mouse hearts are shown in Fig. 5a, b. NMR *E*/*A* ratios and early peak filling velocities were unaltered by age (Fig. 5c, e).

In contrast, we observed a strong influence of age on diastolic filling velocities in mice. Mouse *E*/*A* ratios declined linearly with age from an average of 1.4 ± 0.1 at three months of age to 1.0 ± 0.2 at 2.5 years (Fig. 5d). Peak early diastolic flow velocities declined with age in mice leading to 20% decline between the youngest and oldest cohort (Fig. 5f). Additional diastolic filling parameters can be found in Supplementary Figure S5.

### Naked mole-rats maintain their cardiac functional reserve with advancing age

**Fig. 6:**
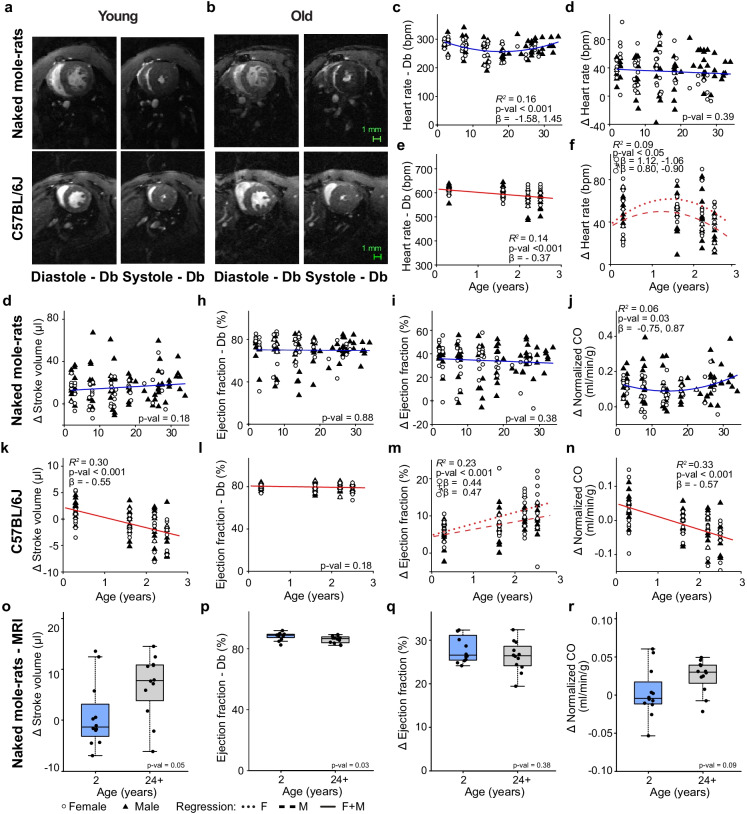
NMR cardiac function under dobutamine stress did not decline with age. **a**, **b** Representative magnetic resonance mid-ventricular short axis images of a young (2 years) and old (32 years) NMR heart as well as a young (0.3 years) and old (2.5 years) mouse heart at end-diastole and end-systole prior to and approximately nine minutes after intraperitoneal infusion of 1.5 mg/kg dobutamine. **c**) NMR heart rate under dobutamine stress showed a quadratic age association (*n*: ♀ = 48 ♂ = 72, Age *β* =  − 1.58, Age^2^ *β* = 1.45, *P* = 2.6E − 5). **d** NMR heart rate changes following dobutamine administration did not change with age (*n*: ♀ = 48 ♂ = 72, Age *β* =  − 0.008, *P* = 0.39). **e** Mouse heart rate under dobutamine stress declined linearly with age (*n*: ♀ = 45 ♂ = 47, Age *β* =  − 0.37, *P* = 2.0E − 4). **f** Mouse heart rate changes following dobutamine administration were quadratically age associated (*n*: ♀ = 45 ♂ = 47, Age ♀*β* = 1.12 Age^2^ ♀*β* =  − 1.06, *P* = 0.33, Age ♂*β* = 0.80 Age^2^ ♂*β* =  − 0.90, *P* = 0.39). **g**, **h** NMR stroke volume change (ultrasound) from baseline and ejection fraction under dobutamine stress did not change with age (*n*: ♀ = 48 ♂ = 72, Age *β* = 0.12, *P* = 0.18 and Age *β* = 0.01, *P* = 0.88). **i** NMR ejection fraction change (ultrasound) following dobutamine administration remained constant with increasing age (*n*: ♀ = 48 ♂ = 72, Age *β* =  − 0.08, *P* = 0.38). **j** NMR normalized cardiac output changes (ultrasound) following dobutamine administration were quadratically age associated (*n*: ♀ = 48 ♂ = 72, Age *β* =  − 0.75, Age^2^ *β* = 0.87, *P* = 0.03). **k** Mouse left ventricular stroke volume changes (MRI) following dobutamine administration declined linearly with age (*n*: ♀ = 45 ♂ = 47, Age *β* =  − 0.55, *P* = 1.8E − 8). **l**, **m** Mouse left ventricular ejection fraction under dobutamine stress did not change with age while ejection fraction changes following dobutamine administration did increase linearly with age (*n*: ♀ = 45 ♂ = 47, Age *β* =  − 0.14, *P* = 0.18 and Age ♀*β* = 0.44, *P* = 2.2E − 3; Age ♂*β* = 0.47, *P* = 8.4E − 4). **n** Mouse normalized cardiac output changes following dobutamine administration declined linearly with age (*n*: ♀ = 45 ♂ = 47, Age *β* =  − 0.57, *P* = 3.0E − 9). **o** Stroke volume changes (MRI) following dobutamine administration were slightly higher in old male NMRs (*n*: ♂ = 12 ♂ = 13, Wilcoxon, *P* = 0.05). **p** Ejection fraction under dobutamine stress (MRI) was higher in young NMRs (*n*: ♂ = 12 ♂ = 13, Wilcoxon, *P* = 0.03). **q**, **r** Ejection fraction and cardiac output changes (MRI) following dobutamine administration were not significantly different between young and old male NMRs (*n*: ♂ = 12 ♂ = 13, Wilcoxon, *P* = 0.38 and *P* = 0.09). Δ: change from unstressed baseline values, Db: dobutamine, scale bars 1 mm

To investigate the link between functional cardiac stress response and aging, we measured left ventricular functional changes following β-adrenergic agonist (dobutamine) infusion in NMRs and mice. Representative diastolic and systolic short-axis MRI images of NMR and mouse hearts are shown in Fig. 6a and b. In both species, left ventricular contractility increased substantially upon dobutamine administration. However, the characteristics of the stress response with increasing age were different.

NMR heart rate under dobutamine stress increased from 234 ± 27 to 270 ± 29 beats per minute(Fig. 6c). These dobutamine induced increases in heart rate did not change with age averaging an increase of 16 ± 11% from baseline (Fig. 6d; Sup. Fig S6a). In contrast, maximum heart rates following dobutamine infusion declined linearly with age in mice from an average of 611 ± 17 bpm at three months to 584 ± 35 bpm at 2.5 years of age (Fig. 6e) leading to 4.2% decline. Heart rate change in response to dobutamine stress was quadratically age associated in mice with reduced heart rate increases for the oldest cohort (Fig. 6f; Sup. Fig. S6f).

NMR stroke volume changes following dobutamine infusion increased slightly with advancing age (Fig. 6g; Sup. Fig. S6d). Ejection fractions increased significantly following dobutamine injection, but ejection fraction increases showed no age dependence in NMRs (Fig. 6h, i). This combined with a quadratic change in body weight led to a quadratic age dependence for normalized changes in cardiac output in NMRs (Fig. 6j; Sup. Fig. S6e). However, there was no difference in normalized cardiac output change following dobutamine infusion between the youngest and oldest NMR cohorts.

Mice showed a linear decline in their ability to increase stroke volumes following dobutamine infusion (Fig. 6k). Although the maximum ejection fraction following dobutamine infusion showed no age dependence in mice, the change from baseline increased linearly with age (Fig. 6m).The inability of mice to increase stroke volumes under stress with advancing age combined with a small decline in the maximum heart rates under these conditions led to linear decline in normalized cardiac output change following dobutamine infusion (Fig. 6n; Sup. Fig. S6j).

As mentioned previously, cardiac function in NMRs was assessed using ultrasound since RFID chips less than four centimeter from the heart interfered with MRI imaging leading to signal loss. To validate dobutamine stress tests in NMRs, MRI was performed on a separate cohort of young and old male NMRs with RFID chips greater than four centimeter from the heart. In agreement with our ultrasound-based measurements, we did not observe a decline in the functional response of young and old NMR hearts following dobutamine administration (Fig. 6o–r; Sup. Fig. S6k-o), confirming the ability of NMRs to maintain their functional cardiac reserve capacity at old age.

These data clearly show that NMRs maintain their ability to increase cardiac output in response to increased demand throughout their expected lifespan, while mice have a declining ability to increase cardiac output as they age.

## Discussion

The importance of age as a risk factor for cardiovascular diseases has long been recognized and has spurred many studies to assess cardiovascular aging in humans and standard laboratory model organisms (mice and rats). However, none of these model organisms exhibit reduced rates of cardiovascular aging. A recent publication showed that naked mole-rats (NMRs) unlike other mammals have a constant mortality hazard with increasing age [11], indicating a remarkable ability to maintain tissue homeostasis and prevent age-associated diseases with Kaplan Meier survival data suggesting that non-breeding NMRs have a median half-life of 19 years and compress morbidity into a very small fraction of lifespan [11]. To test if NMRs can indeed maintain organ homeostasis throughout life, we investigated changes in body composition, bone mineral density, cardiac electrophysiology, cardiac function, and functional reserve capacity across their lifespan and compared them with mice.

We have demonstrated that NMRs maintain body composition and bone mineral density throughout their long lifespan, expanding prior observations of preserved body composition at 20 years of age to 34 years in this study. In contrast to a constant fat mass in NMRs, we observed a significant decline of fat mass in mice older than two years which led to a decline in body weight confirming previous studies [17]. A similar trend has been observed in large cohorts of healthy humans [18, 19].

Similar to the decline in body fat, we observed a significant reduction in mouse femur bone mineral density starting at 2two years of age in line with previous studies analyzing the mechanical strength and composition of femurs in C57BL/6 mice [20, 21]. This progressive loss of bone mineral density is similar to observations in humans [22]. Age associated loss of bone mineral density in humans increases the risk for bone fracture, causing significant disability and secondary cardiovascular events in the elderly. In sharp contrast, naked mole-rats of both sexes maintain bone mineral content and density at ages far in excess of similar-sized mice indicative of stable skeletal homeostasis, bone mineral structure, and mechanical properties [23, 24]. Recent studies revealed that except for the breeding females, NMRs exhibit low rates of bone resorption and bone remodeling [25] which is likely contributing to their stable skeleton.

Another significant factor contributing to cardiac disease are age-associated changes in the cardiac conduction system. The analysis of NMR ECGs did not identify any arrhythmias in young or old animals. This exceptionally well-maintained sinus rhythm of NMRs likely indicates well-maintained cardiomyocyte membrane properties, action potentials, and cardiac conduction. In mice, the number of arrhythmias increases significantly with age in line with previous findings and similar to observations in humans [26, 27]. Cardiac arrhythmias are strongly associated with heart failure, and several large human cohort studies have found baseline arrhythmias to be an independent predictor of all-cause mortality [28–30]. In addition, we observed no difference in QRS duration between old and young NMRs, while mice showed a linear increase with age. Previous studies using different mouse strains did not find increased QRS durations [31, 32], while a telemetry study in Norwegian rats found a linear increase in QRS duration with advancing age [33]. These differences might be due to the larger age range covered in this study. Increased QRS duration is associated with increased risk for congestive heart failure in humans [34]. The observed changes in electrocardiographic features and the prominence of arrhythmias in mice are compatible with increased extracellular matrix formation in aging mice. This may lead to increased conduction parameters (QRS-duration, PR-interval). The decreased diastolic function and the decreased *E*/*A* ratio in mice but not in NMRs also fits this idea (see below).

The short ECG recording time and the use of light anesthesia may have influenced heart rate and conduction parameters and may have suppressed arrhythmogenesis. Thus, the reported arrhythmia incidence is likely an underestimation. Nevertheless, we observed pronounced age-dependent species differences.

Cardiac hypertrophy was clearly detectable in mice but absent in NMRs. In both humans and laboratory rodents, left ventricular hypertrophy, concomitant diastolic dysfunction, and declining contractility are common features of cardiac aging [6, 31, 35, 36] attributed, in part, to enlarged, albeit fewer, cardiomyocytes and less compliant extracellular matrix [35, 37]. While some degree of cardiac hypertrophy can be a beneficial response to increased workload, excessive hypertrophy is a strong predictor for heart failure [38, 39]. Cardiac hypertrophy was also observed in this study as left ventricular wall thickness increased linearly with age in mice indicating cardiac hypertrophy. In contrast to mouse studies, NMR left ventricular wall thickness remained constant through their expected lifespan showing that NMRs, unlike humans and mice, resist age-associated left ventricular hypertrophy during aging. Cardiac hypertrophy is a strong risk factor for diastolic dysfunction but not necessary an indication for it.

Cardiac hypertrophy and changes in the extracellular matrix lead to decreased compliance of the myocardial wall causing diastolic dysfunction; a decline in left ventricular passive filling during early systole and greater dependency on atrial contraction to maximally fill the ventricle prior to contraction. Diastolic dysfunction is a strong predictor for heart failure with preserved ejection fraction in humans [40, 41]. A commonly used measure for diastolic dysfunction is a low early/atrial left ventricular filling velocity (*E*/*A*) ratio. In keeping with prior studies, we observed a constant linear age-associated decline of the *E*/*A* ratios in mice, whereas the *E*/*A* ratios of NMRs did not change with age [8, 13, 42, 43], suggesting that left ventricular compliance is well maintained in NMRs. Several factors affect *E*/*A* ratios limiting its sensitivity. Newer methods such as *E*′/*A*′ and *E*/*E*′ particularly when combined with atrial area measurements provide a more sensitive and robust assessment of diastolic dysfunction [44].

Large age-associated changes have been reported for human cardiac reserve capacity [45]. We therefore examined age-associated changes in cardiac reserve capacity in mice and NMRs following beta adrenergic stimulation with dobutamine. Dobutamine administration increased heart rates and contractility in both species, but there was a decline in the chronotropic response in older mice compared to young mice. A decreasing ability to increase heart rates following beta adrenergic stimulation in mice has been reported previously [46], and similar effects have been observed in humans [45, 47–49]. While beta adrenergic stimulation led to a small increase in stroke volume in young mice, it reduced stroke volumes in older mice leading to a linear decline in normalized cardiac output with advancing age. Our observations in young mice agree with a previous cardiac MRI study [50], but MRI data for older mice is missing in the literature. Similar to our observation in mice a substantial decline in cardiac reserve capacity with advancing age has been observed in humans [45, 47–49]. In contrast to mice and humans, dobutamine-induced increases in stroke volume and cardiac output did not change with age in NMRs indicating preserved cardiac reserve capacity with advancing age.

We relied primarily on ultrasound to measure cardiac parameters in NMRs as all our NMRs are identified by RFID chips, implanted at 90 days in the sub scapular region. These chips often migrate and are found within 3–4 cm from the heart leading to significant signal drop-out on gradient-echo-based MRI. Cardiac MRI is the gold standard for functional assessments in rodents and humans since it does not rely on any geometric assumptions offering full three-dimensional coverage and low variability [51, 52]. The higher variability of ultrasound-based measurements lowers the ability to detect small changes and makes comparisons to MRI based measurements difficult. We consequently performed cardiac MRI on a separate cohort of young and old NMRs whose RFID chips were sufficiently distant from the heart. This analysis showed that NMR cardiac function at rest and under stress does not change with age. It also showed that NMR ejection fraction at rest is approximately 60% and dobutamine increases cardiac output by roughly 10% which is closer to what has been observed in mice than a prior study suggested [53]. The less variable and more precise MRI study confirmed our ultrasound-based findings that NMR cardiac function at rest and under stress does not change with age.

Our ability to detect age-associated changes is limited by two factors: First, the cross-sectional design of our study leads to higher biological variability compared to a longitudinal study where individual baselines can be used for analysis. However, with a maximum lifespan > 38 years in NMRs, longitudinal studies would take decades to complete. Second, the coefficient of variation for the different assays used together with the sample size determines the minimum age-associated change which can be detected. For example, the coefficient of variation for bone mineral density measurements was 2.9% with group sizes *n* > 24 (assuming no sexual dimorphism) limiting the detectable relative change to 2.4% for a two-point comparison. Using the same assumptions with coefficient of variations for MRI-based stroke volume, ejection fraction, and posterior wall thickness measurements of 12.1, 6.1, and 5.9, respectively, we can expect to detect relative changes greater than 10, 5, and 4.9%. There is also a possibility that differences in response to anesthetic gases between young and old animals affect measurements despite standardized temperatures and breathing rates for data acquisition.

## Conclusion

This study demonstrates that unlike mice that exhibit pronounced declines in body composition and cardiac function commencing shortly after sexual maturity, NMRs can maintain tissue homeostasis throughout their four-decade long maximum lifespan. Furthermore, NMRs do not show any signs of diastolic dysfunction or cardiac hypertrophy and maintain similar functional cardiac reserve capacity at advanced age to that exhibited when young adults, at the prime of life. Collectively, these data reveal that the naked mole-rat provides a proof-of-concept that age-related declines in body composition and cardiac function are not inevitable. Elucidating these mechanisms may lead to the discovery of therapies to reduce the burden of age-associated cardiovascular pathology, morbidity, and mortality and thereby enhance quality of life in older humans.

## Supplementary Information

Below is the link to the electronic supplementary material.

Supplementary file1 (DOCX 2105 KB)

## Data Availability

Raw data is available upon request.

## Code availability

Custom processing scripts are available upon request.
