# Supplementary material for: Naked mole-rats maintain cardiac function and body composition well into their fourth decade of life
Source: GeroScience. 2022 Feb 2;44(2):731–46. doi: 10.1007/s11357-022-00522-6 (PMC9135933; doi:10.1007/s11357-022-00522-6)
Supplement: Supplementary file 1 — Supplementary file1 (DOCX 2105 KB) [file 11357_2022_522_MOESM1_ESM.docx]

**Supplemental Figure S1. Neither NMR body fat mass nor femoral bone mineral content**  **changes with age.**

**(a)** NMR fat mass did not change with advancing age (n: ♀=48 ♂=72, Age β=-0.10, P=0.28). **(b)** Mouse fat mass changed quadratically with age (n: ♀=62 ♂=70, Age ♀β=3.25 Age2 ♀β=-2.87 P<=9.5E-14, Age ♂β=3.65 Age^2^ ♂β=-3.52 P<=2.2E-16). **(c)** NMR bone mineral content showed a small linear increase with advancing age (n: ♀=48 ♂=72, Age β=0.19, P=0.03). **(d)** Mouse bone mineral content showed a strong age dependence (n: ♀=62 ♂=70, Age ♀β=1.41 Age2 ♀β=-0.92 P<=3.7E-5, Age ♂β=-0.26 Age2♂β=0.90 P<=6.2E-9).

**Supplemental Figure S2. NMR P-wave amplitude and PQ interval do not change with age.**

**(a)** NMR P-wave amplitude did not change with age (n: ♀=48 ♂=72, Age β=-0.07, P=0.41). **(b)** Mouse P-wave amplitude showed a small quadratic age dependence (n: ♀=62 ♂=70, Age β=0.95Age^2^ β=-0.82 P=0.04). **(c)** NMR R-wave amplitude decreased with age (n: ♀=48 ♂=72, Age β=-0.19, P=0.03). **(d)** Mouse R-wave amplitude did not change with age (n: ♀=62 ♂=70, Age β=6.7E-4, P=0.99). **(e)** NMR T-wave amplitude did not change significantly with age (n: ♀=48 ♂=72, Age β=0.03, P=0.75). **(f)** In contrast, mouse T-wave amplitude decreased slightly with age (n: ♀=62 ♂=70, Age β=-0.22, P=0.01).

**Supplemental Figure S3. Representative ECG traces from old mice with common arrythmia types.** **(a-c)** Three lead (L1-L3) ECG recordings from a 2.2-year-old male mouse with ventricular premature beats. **(d-f)** Three lead (L1-L3) ECG recordings from a 2.2-year-old male mouse with atrial premature beats. **(g-i)** Three lead (L1-L3) ECG recordings from a 2.2-year-old male mouse with junctional premature beats.

**Supplemental Figure S4. While mouse left ventricular wall thickness increases with age it remained constant in NMRs.**

**(a)** Representative ultrasound 2-chamber long axis images of a young (2.9 years) and old (27.4 years) male NMR heart at end-diastole and end-systole. **(b)** NMR cardiac output (ultrasound, not normalized) did not change with age (n: ♀=48 ♂=72, Age β=0.02, P=0.82). **(c)** Mouse cardiac output (MRI, not normalized) increased linearly with age (n: ♀=47 ♂=49, Age ♀β=0.54, Age ♂β=0.39, P=1.6E-07). **(d-f)** Mouse left ventricular wall thickness at end-diastole increased linearly with age. Examples for AHA segments 7, 9, 11 (n: ♀=47 ♂=49, Age β=0.33, P=0.001, Age β=0.51 P=1.3E-07, and Age β=0.49 P=4.4E-07 respectively). **(g)** Mouse left ventricular mass increased linearly with age (n: ♀=47 ♂=49, Age β=0.74, P<2.2E-16). **(h)** Cardiac output (MRI) of young and old male NMRs was not significantly different (n: ♂=12 ♂=13, Wilcoxon P=0.98). **(I)** Young and old NMR left ventricular mass (MRI) was not significantly different (n: ♂=12 ♂=13, Wilcoxon P=0.69). **(j, k)** Young and old NMR left ventricular wall thicknesses at end-diastole was not significantly different. Examples for AHA segments 9, 10 (n: ♂=12 ♂=13, Wilcoxon P=0.12 and P=0.32). **(l)** Body weights of Young and old male NMR cohorts used for cardiac MRI studies.

**
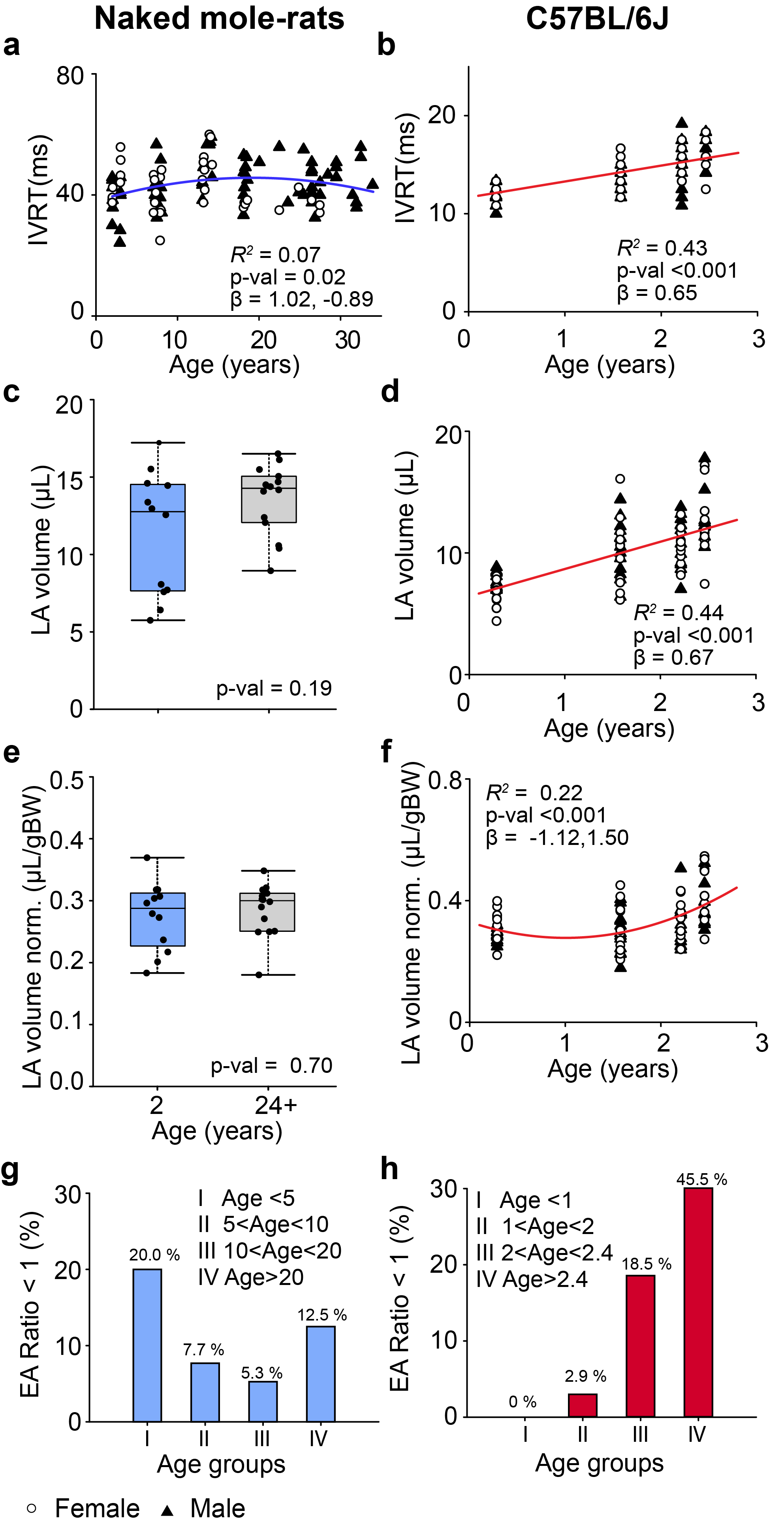
**

**Supplemental Figure S5. IVRT and left atrial volumes increase with age in mice but not in NMRs.**

**(a)** NMR isovolumetric relaxation time (IVRT) although markedly longer than that of the mouse, showed a quadratic age dependency (n: ♀=48 ♂=72, Age β=1.02, Age^2^ β=-0.89, P=0.02). **(b)** Mouse IVRT increased linearly with age (n: ♀=62 ♂=70, Age β=0.65, P<0.001). **(c)** MRI derived left atrial volumes (biplane method) were similar for young and old NMRs (1.9±0.1 and 28.2±2.3 years; n: ♂=12 ♂=13, Wilcoxon P=0.19). **(d)** MRI derived mouse left atrial volumes (biplane method) increased linearly with age (n: ♀=48 ♂=72, Age β=0.67, P<0.001). **(e)** NMR left atrial volumes normalized to body weight did not differ between young and old animals (n: ♂=12 ♂=13, Wilcoxon P=0.70). **(f)** Mouse left atrial volumes normalized to body weight showed a quadratic age dependent increase (n: ♀=48 ♂=72, Age β=-1.12, Age^2^ β=1.50, P<0.01). **(g)** Although young NMRs had E/A ratios <1, their frequency did not increase with age (n: ♀=48 ♂=72). **(h)** All young mice had E/A ratios >1 but with advancing age an increasing number of mice had E/A ratios <1 (n: ♀=62 ♂=70).

**Supplemental Figure S6. NMR cardiac stress responses do not change with age.**

**(a)** NMRs heart rate change (in %) following dobutamine administration (Db) showed no age dependence (n: ♀=48 ♂=72, Age β=-0.06, P=0.51). **(b)** NMR end-diastolic volume (EDV, ultrasound) following dobutamine administration did not change with age (n: ♀=48 ♂=72, Age β=0.08, P=0.36). **(c)** NMR end-systolic volume (ESV, ultrasound) under dobutamine-induced stress did not change with age (n: ♀=48 ♂=72, Age β=0.02, P=0.83). **(d)** NMR stroke volume (ultrasound) under dobutamine-induced stress did not change with age (n: ♀=48 ♂=72, Age β=0.13, P=0.16). **(e)** NMR cardiac output (CO) following dobutamine administration did not change with age (n: ♀=48 ♂=72, Age β=0.07, P=0.48). **(f)** Mouse heart rate changes (in %) following dobutamine administration showed a weak quadratic age dependence (n: ♀=45 ♂=47, Age ♀ β=1.11 Age^2^ ♀β=-1.04 P=0.33, Age ♂β=0.79, Age2 ♂β=-0.84, P=0.48). **(g)** Mouse EDV (MRI) under dobutamine stress increased linearly with age (n: ♀=45 ♂=47, Age ♀β=0.59, Age ♂β=0.38, P=3.3E-7). **(h)** Mouse ESV (MRI) under dobutamine-induced stress increased linearly with age (n: ♀=45 ♂=47, Age ♀β=0.47 P=1.1E-3, Age ♂β= 0.31, P=0.03). **(i)** Mouse stroke volume under dobutamine-induced stress showed a linear increase with age (n: ♀=45 ♂=47, Age♀ β=0.54, P=1.1E-4, Age♂ β=0.38, P=8.7E-3). **(j)** Mouse cardiac output under dobutamine stress showed a linear age dependence (n: ♀=45 ♂=47, Age ♀β=0.52 P=2.7E-4, Age ♂β=0.23 P=0.12). **(k)** NMR heart rate changes following dobutamine administration were not significantly different between young and old male NMRs (n: ♂=12 ♂=13, Wilcoxon P=0.24). **(l, m)** NMR left ventricular end-diastolic and end-systolic volumes (MRI) were not significantly different between young and old NMRs (n: ♂=12 ♂=13, Wilcoxon P=0.19 and P=0.27). **(n)** Stroke volumes (MRI) under dobutamine stress were slightly higher in old NMRs (n: ♂=12 ♂=13, Wilcoxon P=0.05). **(o)** Cardiac output under dobutamine stress was not significantly different between young and old NMRs (n: ♂=12 ♂=13, Wilcoxon P=0.17 ). ∆: change from unstressed baseline value.
